# QuantLaneNet: A 640-FPS and 34-GOPS/W FPGA-Based CNN Accelerator for Lane Detection

**DOI:** 10.3390/s23156661

**Published:** 2023-07-25

**Authors:** Duc Khai Lam, Cam Vinh Du, Hoai Luan Pham

**Affiliations:** 1 Computer Engineering Department, University of Information Technology, Ho Chi Minh City 700000, Vietnam; 18521646@gm.uit.edu.vn; 2 Vietnam National University, Ho Chi Minh City 700000, Vietnam; 3 Graduate School of Information Science, Nara Institute of Science and Technology, Nara 630-0192, Japan; pham.hoai_luan.ox7@is.naist.jp

**Keywords:** convolutional neural network, lane detection, QuantLaneNet, hardware architecture, FPGA

## Abstract

Lane detection is one of the most fundamental problems in the rapidly developing field of autonomous vehicles. With the dramatic growth of deep learning in recent years, many models have achieved a high accuracy for this task. However, most existing deep-learning methods for lane detection face two main problems. First, most early studies usually follow a segmentation approach, which requires much post-processing to extract the necessary geometric information about the lane lines. Second, many models fail to reach real-time speed due to the high complexity of model architecture. To offer a solution to these problems, this paper proposes a lightweight convolutional neural network that requires only two small arrays for minimum post-processing, instead of segmentation maps for the task of lane detection. This proposed network utilizes a simple lane representation format for its output. The proposed model can achieve 93.53% accuracy on the TuSimple dataset. A hardware accelerator is proposed and implemented on the Virtex-7 VC707 FPGA platform to optimize processing time and power consumption. Several techniques, including data quantization to reduce data width down to 8-bit, exploring various loop-unrolling strategies for different convolution layers, and pipelined computation across layers, are optimized in the proposed hardware accelerator architecture. This implementation can process at 640 FPS while consuming only 10.309 W, equating to a computation throughput of 345.6 GOPS and energy efficiency of 33.52 GOPS/W.

## 1. Introduction

The rapid development of autonomous vehicles requires continuous improvement in the ability to perceive the environment around the vehicle. One of the main perception modules is vision-based lane detection. Traditionally, the task of lane detection has been attempted by using hand-crafted algorithms, such as color-based [1,2] or Hough Transform [3,4]. Despite being able to detect at rapid speed, these methods prove to be limited as they are not robust enough against the vast variations in real-world road scenes, such as poorly marked lanes, shadows, low illumination, or occlusion. More recently, this problem has been mainly shifted towards the field of deep learning, more specifically Convolutional Neural Networks (CNNs), as they are capable of outstanding accuracy across numerous applications. Wen et al. [5] propose a unified viewpoint transformation (UVT) method that transforms the camera viewpoints of different datasets into a common virtual world coordinate system to tackle the dataset bias between the training and test datasets to improve lane detection performance. Li et al. [6] presents a framework of two types of deep neural networks to learn the structures for visual analytics. The first is the convolutional neural network (CNN) to build global objects (location and orientation). The second is the recurrent neural network (RNN) to detect local visual lanes in a traffic scene. This work leads to superior recognition performance. Kim et al. [7] present a method that combines the random sample consensus (RANSAC) algorithm with the convolutional neural network (CNN) to improve the accuracy of traffic lane detections. RetinaNet [8] proposes a detection and classification method for various types of arrow markings and bike markings on the road in various complex environments using a one-stage deep convolutional neural network (CNN). LkLaneNet [9] proposes a novel multi-lane detection method called Large Kernel Lane Network to detect multiple lanes accurately and efficiently in challenging scenarios. ZF-VPGNet [10] builds a multi-task learning network consisting of multi-label classification, grid box regression, and object mask. This work obtains the correct results and achieves high accuracy and robustness. Alam et al. [11] propose a lane detection method using the microlens array-based light field camera image that uses the additional angular information. This approach increases accuracy in challenging conditions, such as illumination variation, shadows, false lane lines, and worn lane markings. Chao et al. [12] combine a deep convolutional neural network to classify the lane images at the pixel level with the Hough transform to determine the interval and the least square method to fit lane marking. This approach helps to improve the accuracy performance to 98.74 %. Zhang et al. [13] use a monocular camera to study a lane-changing warning algorithm for highway vehicles based on deep learning image processing. This system improves vehicle driving safety in a low-cost manner. SUPER [14] proposes a novel lane detection system consisting of a hierarchical semantic segmentation network as the scene feature extractor and a physics-enhanced multi-lane parameter optimization module for lane inference. This approach provides better accuracy.

These above approaches show their effectiveness in improving the accuracy performance; however, the calculation costs are incredibly high because they utilize a heavy deep learning network. These networks need to be simplified to apply this application on embedded devices with limited resources. LLDNet [15] introduced a lightweight CNN model based on an encoder–decoder architecture that makes it suitable for being implemented in embedded devices. Podbucki et al. [16] present an NVIDIA Jetson Xavier AGX embedded system to process video sequences for lane detection. Jayasinghe et al. [17] propose a simple, lightweight, end-to-end deep learning-based framework coupled with the row-wise classification formulation for fast and efficient lane detection. This system is implemented on an Nvidia Jetson AGX Xavier embedded system to achieve a high inference speed of 56 frames per second (FPS). Liu et al. [18] propose a lightweight network, named as LaneFCNet, combined with the conditional random field for lane detection to reduce processing time. Hassan et al. [19] discuss an improved CNN-based detection system for autonomous roads to identify potholes, cracks, and yellow lanes. This system is implemented and verified on the Jetson TX2 embedded system. Due to the limitations of processing power, memory, and other resources of the embedded devices, these approaches are hard to reach in real-time for their processing.

To speed up processing for real-time applications, the hardware platforms are used to design and implement. Martin et al. [20] present an algorithm for detecting lane markings from images. It is designed and implemented in Field Programmable Gate Arrays (FPGA) technology on Zynq-7000 System-on-Chip (SoC). The algorithm uses traditional computer vision techniques to obtain lane markings and detect driving lanes. Kojima et al. [21] presents an autonomous driving system consisting of lane-keeping, localization, driving planning, and obstacle avoidance that are implemented as software in the embedded processor on FPGA. Wang et al. [22] propose a detailed procedure that helps guide the performance optimization of parallelized ADAS applications in an FPGA-Graphics Processing Unit (GPU) combined heterogeneous system. Kamimae et al. [23] develop an SoC FPGA based on the Helmholtz Principle to control unmanned mobile vehicles for the FPGA design competition. Peng et al. [24] build a multi-task learning framework for lane detection, semantic segmentation, 2D object detection, and orientation prediction on FPGA. The performance on FPGA is optimized by software and hardware co-design to achieve 55 FPS. A CNN for drivable region segmentation from a LiDAR sensor called ChipNet [25,26] is designed and implemented on FPGA, which achieves 79.43 FPS. Utilizing the scheme presented in [27], RoadNet-RT [28] designs and implements a CNN on FPGA using 8-bit quantization. Their FPGA implementation achieves 197.7 FPS. These approaches obtain good performance in processing speed; however, the trade-off among processing speed, accuracy, hardware resources, and power consumption is not fully discussed in these studies.

The segmentation is a common strategy for lane detection works. This strategy typically outputs a pixel map with an identical size to the input RGB image, where each pixel is classified into a different class, such as roads, cars, pedestrians, etc. U-Net [29] presents a full convolution network and training strategy that relies on the strong use of data augmentation to work with very few training images and yields more precise segmentations in the biomedical field. ENet [30] proposes a novel deep neural network architecture on embedded systems to perform real-time semantic segmentation. SegNet [31] presents a segmentation engine consisting of an encoder network and a corresponding decoder network followed by a pixel-wise classification layer. The decoder uses pooling indices computed in the max-pooling step of the corresponding encoder to perform non-linear upsampling. This eliminates the need for learning to upsample. The upsampled maps are sparse and are then convolved with trainable filters to produce dense feature maps. This design achieves good segmentation performance. Zou et al. [32] propose a hybrid deep architecture by combining the convolutional neural network (DCNN) and the recurrent neural network (DRNN), where the DCNN consists of an encoder and a decoder with fully-convolution layers, and the DRNN is implemented as a long short-term memory (LSTM) network. The DCNN abstracts each frame into a low-dimension feature map, and the LSTM takes each feature map as a full-connection layer in the timeline and recursively predicts the lane. The LSTM is found to be very effective for information prediction and significantly improves the performance of lane detection in the semantic segmentation framework. Davy et al. [33] design a branched, multi-task network for lane instance segmentation, consisting of a lane segmentation branch and a lane embedding branch that can be trained end-to-end. The lane segmentation branch has two output classes, background or lane, while the lane embedding branch further disentangles the segmented lane pixels into different lane instances. This approach can handle various lanes and cope with lane changes.

**Figure 1:**
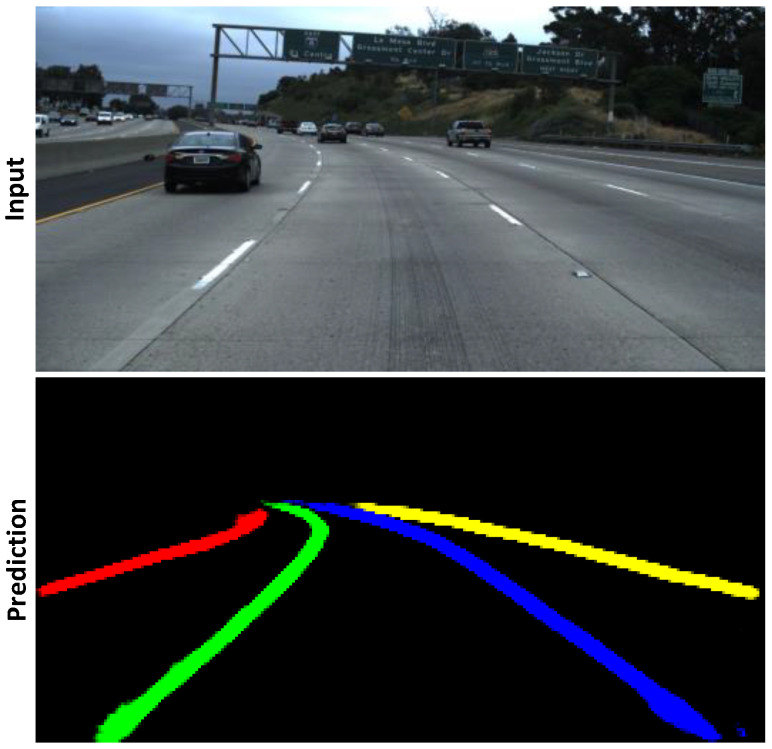
Lane instance segmentation: each pixel in the prediction is classified into a separate class, denoted by a different color. While visually informative, this formulation requires much post-processing to extract geometric information.

While methods utilizing the segmentation technique yield accurate results, they suffer from low efficiency. For example, lane markings are slim and continuous lines that do not require clusters of dense pixels to represent. Moreover, segmentation requires significant post-processing to extract geometric information about the lane lines, which introduces further inefficiency. Figure 1 illustrates lane segmentation. Recent studies attempted to solve this problem by substituting dense pixel segmentation for sparser or more descriptive representations. Inspired by the Region Proposal Network (RPN) of Faster R-CNN [34], Line-CNN [35] utilizes the line proposal unit (LRU) to predict lanes using predefined straight rays. PolyLaneNet [36] attempts to frame lane detection as a polynomial regression problem. UFAST [37] proposes a row-wise formulation, where the output is a series of horizontal locations corresponding to predefined row anchors. This work puts emphasis on obtaining real-time speed. Later, CondLaneNet [38] adds a vertical range and offset map to improve the row-wise formulation. Another approach is PINet [39], which predicts sparse key points from the input image, which is then clustered into different instances by an embedding branch. However, the runtime results of these models are evaluated on power-hungry GPUs. Despite obtaining state-of-the-art accuracy, the massive power consumption of these GPUs proves impractical for power-constrained systems on cars.

In this work, we propose a simple and efficient lane representation format alongside a lightweight lane detection CNN, named QuantLaneNet, to achieve real-time processing at the low cost of hardware design resources and power consumption. Meanwhile, the accuracy of traffic lane detection is relatively close to the related works. The contributions of this paper are summarized as follows:

- To efficiently represent the geometric information of lane markings, we propose a lightweight format that only requires two small arrays for minimum post-processing instead of segmentation maps. Using our format as the shape for the output, we propose a simple and lightweight CNN for the task of lane detection. The model consists of three encoder stages to extract features from the input image and two output branches that produce the two matrices which make up our proposed lane representation format. Our model only contains 102k parameters and achieves 348.34 FPS on the NVIDIA Tesla T4 GPU.
- To optimize processing speed and power consumption, a corresponding hardware accelerator is designed and implemented on Xilinx Virtex-7 VC707 FPGA as a Peripheral Component Interconnect Express (PCIe) device. By optimizing the techniques, such as data quantization to reduce data width down to 8-bit and exploring various loop-unrolling strategies for different convolution layers, and pipelined computation across layers, our architecture achieves very high throughput while consuming very little power compared to other studies. Using the verification system described in Section 5.2, our FPGA system achieves 640 FPS while consuming only 10.309 Watts (W), which equates to a throughput of 345.6 giga operations per second (GOPS) and an energy efficiency of 33.52 GOPS/W. The trade-off in accuracy due to data quantization is negligible, and the overall accuracy is relatively close to that of the related works.

The rest of the paper is organized as follows. Section 2 presents the background of this work. Section 3 presents the proposed lane representation format and CNN model, along with training and evaluation details. Section 4 describes several optimizations for designing a hardware accelerator, and the implementation of the final design is presented in Section 5. Finally, Section 6 concludes this paper.

## 2. Background

### 2.1. Convolutional Neural Networks

Convolutional neural networks (CNNs) have gained prominence in the field of computer vision due to their ability to capture spatial patterns in images. For images, their visual meaning comes from local and global patterns instead of the values of each individual pixel that makes up the image. By applying sliding windows (called kernels) on the input image, CNNs can capture local patterns from each group of pixels. During training, the values of the kernels (called parameters or weights) can be “learned” so that each convolutional layer can extract the correct features relative to its purpose, as opposed to handcrafted kernels that can be susceptible to variations in the environment.

Each convolutional layer can be configured differently by changing its kernel size, padding, stride, and dilation. A larger kernel size can give the layer a bigger depth of field but will increase the number of parameters dramatically. Padding is used to add border pixels to the input to preserve the output size. Stride determines the step size of the sliding kernel. It affects the receptive field of the layer and will typically reduce the size of the output, unless padding is applied. Dilation adds gaps in the kernel so that the kernel’s depth of field can be increased without adding more parameters. All of these values can be used in combination with each other for many reasons, such as manipulating the size of the output or emphasizing local or global patterns, among others.

A convolutional layer is typically followed by a non-linear activation function, such as ReLU or sigmoid. In a supervised training context, a dataset with inputs and human-labeled correct outputs is used. The inputs are fed through the model, and a “score”, formally referred to as the loss, is calculated from the outputs and the labels using the loss function. Through backpropagation, the parameters within the model are incrementally adjusted to minimize the loss value through many training iterations. Based on many elements such as purpose, the shape of the output, model’s architecture, etc., the loss function will have to be carefully chosen, or in many cases, developed, to achieve the best accuracy.

### 2.2. Data Quantization

Data quantization is a commonly used technique with the main aim of decreasing the quantity of discrete values present in a given system. The method involves representing a continuous or high-resolution range of values with a limited range of discrete values, typically in a much lower bit width. The utilization of this technique extends to diverse fields, including signal processing, data compression, and machine learning. Within the domain of machine learning, it assumes a pivotal role in the optimization of neural network inference. Instead of retaining the floating-point values used in training, the technique converts these values into a low-precision representation to use in inference. This not only reduces the memory footprint of the system but also decreases processing time and complexity significantly.

Despite the numerous advantages of quantization, the data precision of the system will inevitably be compromised. The amount of memory/speed to precision trade-off will have to be evaluated by the designer to best fit the requirements of each specific system.

### 2.3. Dataset and Accuracy Formulation

The open-source TuSimple dataset [40], which annotates lanes as the sets of horizontal coordinates corresponding to fixed vertical coordinates. TuSimple dataset includes 3626 video clips, 3626 annotated frames for the training phase, and 2782 video clips for the testing phase. These videos cover the good and medium weather conditions at different daytime. They include 2-lane, 3-lane, and 4-lane roads in different traffic conditions. The main evaluation metrics for TuSimple are accuracy, false positive, and false negative. Accuracy is defined by Equation (1), where $C_{clip}$ is the number of correct points in the frame and $S_{clip}$ is the number of requested points:

(1) $$accuracy=\frac{\sum_{clip}C_{clip}}{\sum_{clip}S_{clip}}$$

False positive (FP) and false negative (FN) are defined by Equations (2) and (3), respectively, where $F_{pred}$ is the number of wrongly predicted lanes, $N_{pred}$ is the number of all predicted lanes, $M_{pred}$ is the number of missed ground-truth lanes in the prediction and $N_{gt}$ is the number of all ground-truth lanes:

(2) $$FP=\frac{F_{pred}}{N_{pred}}$$

(3) $$FN=\frac{M_{pred}}{N_{gt}}$$

## 3. Proposed Network

### 3.1. Lane Representation Format

**Figure 2:**
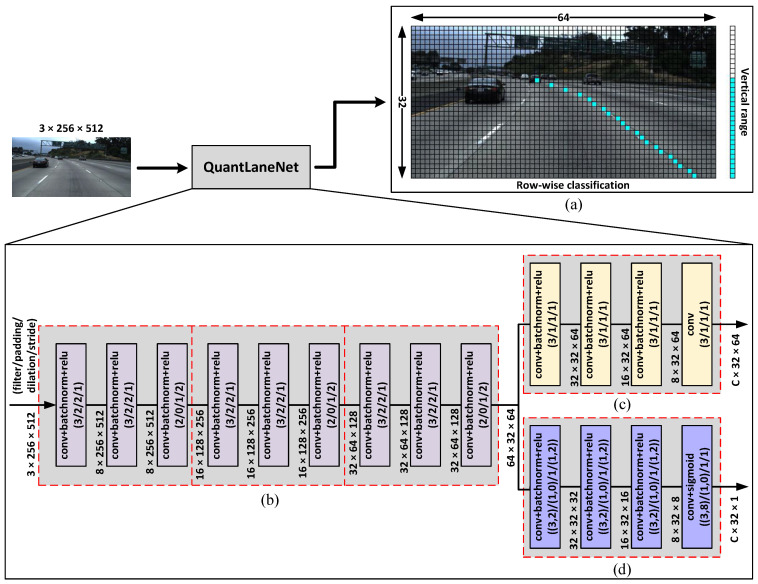
Proposed model overall architecture (C denotes the number of lanes): (**a**) Lane representation format, (**b**) Encoder stages, (**c**) Row-wise classification branch, and (**d**) Vertical range branch.

In this paper, we propose an efficient row-wise formulation to minimize post-processing, as shown in Figure 2a. The goal of our proposed formulation is to use a much smaller amount of data compared to segmentation maps. This approach minimizes the amount of post-processing required while still providing sufficient geometric information on the lane markings. To achieve this, our model predicts lanes as a small group of key points sampled at regular vertical intervals. These key points are represented in the output as a grid of size 32×64 called row-wise classification and a column of size 32×1 called vertical range. Each cell in the vertical range has a value between 0 and 1, and cells that are ≥0.5 denote lane presence in the corresponding classification rows. Once the classification rows are identified, the horizontal locations of the lane can be determined by the cells with the highest value in each row. This pair of arrays is replicated for every lane predicted by our model. Additionally, we also experimented with the offset map described by CondLaneNet [38] and PINet [39]. The results of our experimentations are discussed in Section 3.5.

With this formulation, an RGB image of size 3×256×512 can be represented by as little as a 4×32×64 and a 4×32×1 array for 4 lanes. Compared to the size of the input image, the output is over 47 times smaller.

### 3.2. Network Architecture

Utilizing our formulation, we propose QuantLaneNet, a lightweight CNN for the task of lane detection. Figure 2 shows the overall architecture of our model. The model consists of 3 encoder stages to resize the input down to 32×64 to reduce inference time on the subsequent layers. The resized feature maps are then fed to 2 parallel output branches that predict the row-wise classification and vertical range. The fundamental unit block of our model is a sequence of 3 layers, convolution, batch normalization [41], and ReLU, which will be denoted as “conv+bn+relu” for the remainder of this paper.

Each encoder stage consists of 3 “conv+bn+relu” layers to half the height and width of the input while doubling the number of channels. Dropout [42] with p=0.2 is applied to every convolution layer during training to minimize overfitting. Figure 2b shows the structure of the 3 encoder stages in our model.

The row-wise classification branch, shown in Figure 2c, contains 3 “conv+bn+relu” layers and a convolution layer with linear activation at the end. Through each layer, the input size is maintained while the number of channels is halved. The absence of an activation function at the last layer is to maintain the difference between values on the same row. To elaborate, because only the cell with the highest value in each row is needed, applying a non-linear activation would compress the difference between the highest cell and the rest of the cells. With the lower precision used by our hardware accelerator, such compressed difference will be lost. In training, sigmoid is applied to the output of the classification branch to be compatible with cross-entropy loss.

In the vertical range branch, shown in Figure 2d, convolution layers are chosen as opposed to dense layers like UFAST [37] and CondLaneNet [38]. Dense layers are not preferable for hardware designs since they require a large number of weights. The vertical branch shares a similar structure to the classification branch, with 3 “conv+bn+relu” layers followed by a “conv+sigmoid” layer at the end. Through each layer, the width of the input is decreased by half so that the final output only has a width of 1, as presented in our proposed formulation. Also, following the proposed formulation, the sigmoid function is chosen for the last layer to keep the output values between 0 and 1.

Similar to the encoder stages, dropout with p=0.2 is also applied to every convolution layer to minimize overfitting, except for the last layer of both branches.

### 3.3. Loss Functions and Training Details

For training, two loss functions are applied to each output branch:

1. Classification loss: For the row-wise classification branch, the loss is calculated for each row separately and subsequently summed up. Equation (4) shows the classification loss, where *C* is the number of lanes, *h* is the classification height, $cls_{i,j,:}$ is the classification prediction at row *j* lane *i*, $cls_{i,j,:}^{*}$ is the corresponding ground truth and $L_{CE}$ denotes the cross-entropy loss:
  (4) $$L_{cls}=\sum_{i=1}^{C}\sum_{j=1}^{h}L_{CE}\left(cls_{i,j,:},cls_{i,j,:}^{*}\right)$$
2. Vertical loss: Similar to classification loss, the vertical loss is the sum of all cross-entropy loss on each lane. Equation (5) shows the vertical loss, where *C* is the number of lanes, $vert_{i,:}$ is the vertical prediction at lane *i*, $vert_{i,:}^{*}$ is the corresponding ground truth and $L_{CE}$ denotes the cross-entropy loss:
  (5) $$L_{vert}=\sum_{i=1}^{C}L_{CE}\left(vert_{i,:},vert_{i,:}^{*}\right)$$

The total loss for training is the weighted sum of the above loss terms. Equation (6) shows the total loss:

(6) $$L_{total}=L_{cls}+L_{vert}$$

All input images are resized to 256×512 and normalized from RGB values between 0 and 255 to values between 0 and 1. The number of lanes is fixed to 4 as the number of lanes present in the majority of images in the dataset. Labels are converted from the format of the dataset to our proposed format. The source code is written in PyTorch [43], a deep learning framework for the Python programming language [44]. The model is trained using Adam optimizer [45] and the learning rate is kept 1e-3 as default. Due to the limited memory of the training GPU, the batch size is set to 16. Finally, we trained the model for 150 epochs, and the epoch with the highest accuracy is chosen as the final model.

### 3.4. Accuracy Evaluation

**Figure 3:**
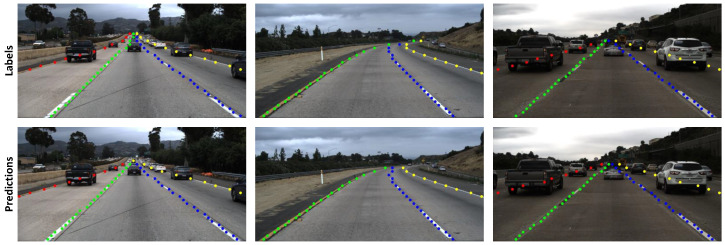
Some results for TuSimple dataset.

We trained and evaluated our model using the open-source TuSimple dataset. To evaluate our model, the predictions are converted back into TuSimple’s annotation format. Framerate is sampled while running on an NVIDIA Tesla T4. Results and comparison details are shown in Table 1. The results show our design takes only 0.540 giga floating point operations (GFLOPs), much smaller than the related works. In addition, our processing speed (384.3 FPS) is also much greater than the related works. Meanwhile, the accuracy (93.53 %) is slightly lower than the related works. The demonstrations of some Tusimple video samples are shown in Figure 3. The results show that the prediction outputs are matched to the labels.

### 3.5. Experiments with Offset Map

**Figure 4:**
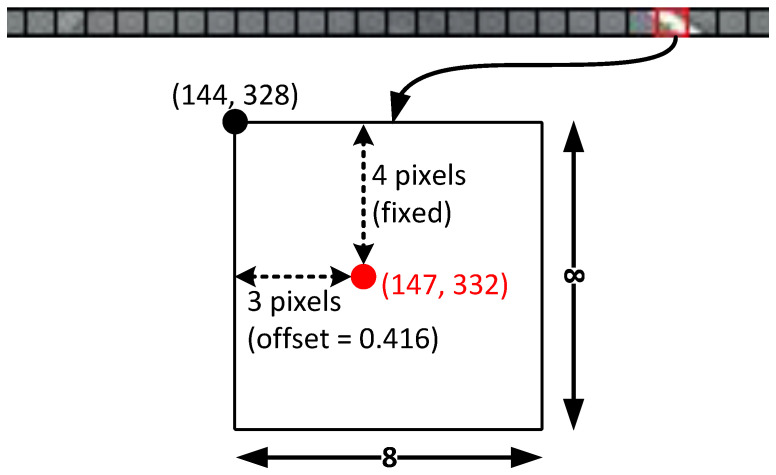
Refinement via offset map. The coordinates of the point after refinement are indicated by the red color.

**Figure 5:**
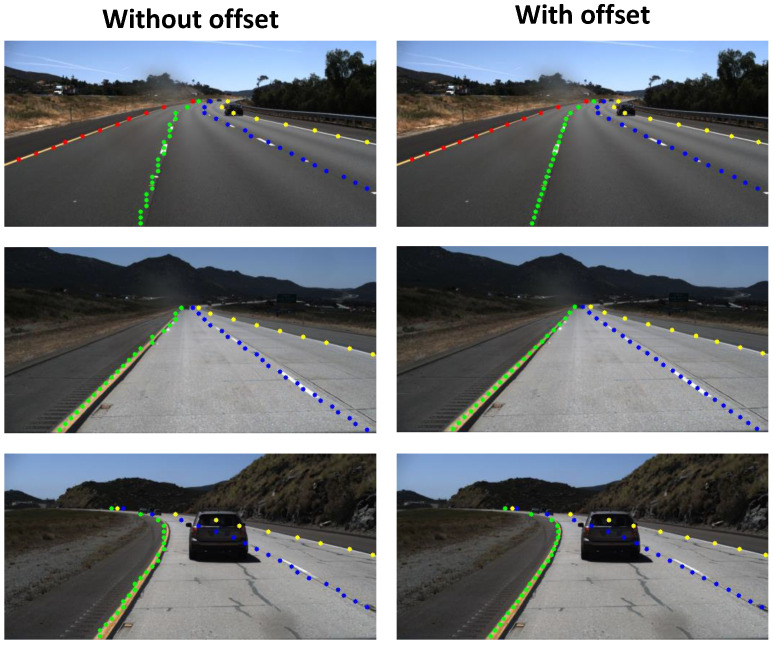
The addition of an offset map can help smooth out the output points.

As row-wise classification is a sparse representation of the input image, one of its points actually covers a large number of pixels. For the input and output size of our model, a point in classification covers an 8×8 grid in the input. To further refine the locations of the point in row-wise classification, PINet [39] and CondLaneNet [38] used the offset map. This map is a matrix of the same size as row-wise classification, with each cell having a value between 0 and 1. For each identified point in row-wise classification, the corresponding value in the offset map (between 0 and 1) is mapped to the width of the grid to pinpoint the exact horizontal coordinate of the point. The working principle of the offset map is illustrated in Figure 4, while Figure 5 shows a visualization of the output with the offset map applied.

Figure 5 shows that the offset map can help visually smooth out the lane coordinates. However, the effect this output has on overall accuracy still needs to be evaluated. To determine whether the offset map is required in our model, we added another output branch for the offset output. This branch is identical in structure to the row-wise classification branch (Figure 2c), except that the last layer uses the sigmoid function for activation to keep the values between 0 and 1.

This branch is trained using the L1 loss. Because ground truth does not exist at cells that lane lines do not pass through, we follow PINet by ignoring these cells when calculating loss. Equation (7) shows the offset loss, where $G_{e}$ is the group of points where row-wise classification exists in the ground truth, $c_{x}$ is the offset prediction, $c_{x}^{*}$ is the corresponding ground truth and ${∥.∥}_{1}$ denotes the L1 loss.

(7) $$L_{offset}=\sum_{c_{x}\in G_{e}}∥c_{x}-c_{x}^{*}{∥}_{1}$$

To train this version of our model, the offset loss is added to the total loss, as shown in Equation (8).

(8) $$L_{total}=L_{cls}+L_{vert}+L_{offset}$$

Finally, the accuracy comparison between the base model and the model with the offset map added is presented in Table 2. It can be seen that while an offset map can bring visual improvements to the output, its impact on accuracy is minimal, raising only 0.02% in accuracy. This is because while the coordinates of the points without offset have a tendency to zigzag along the line, they still run across the line, thus preserving the geometry of the curve. Because of this, the offset map is ultimately excluded from our final model, as the accuracy impact is not worth the trade-off in additional computations.

## 4. Proposed Hardware Architecture

### 4.1. Data Quantization

In specific application hardware designs, efficient fixed-point arithmetic is usually preferred to floating-point, due to the significant resource utilization required by floating-point. Data quantization is a commonly used technique that is also natively supported by PyTorch. In this work, post-training static quantization to 8-bit is utilized.

Accuracy impact after quantization is shown in Table 3. Quantizing weights and activations down to 8-bit carries a 0.06% accuracy loss compared to the 32-bit floating point, which is determined to be acceptable, especially since such data width enables significant hardware optimization. More specifically, as presented in [27], each Xilinx’s DSP48E2 core can perform two 8-bit multiplications simultaneously, albeit with a common multiplicand. Thus, 8-bit arithmetic has the potential to double the throughput compared to wider data widths.

### 4.2. Overall Design

**Figure 6:**
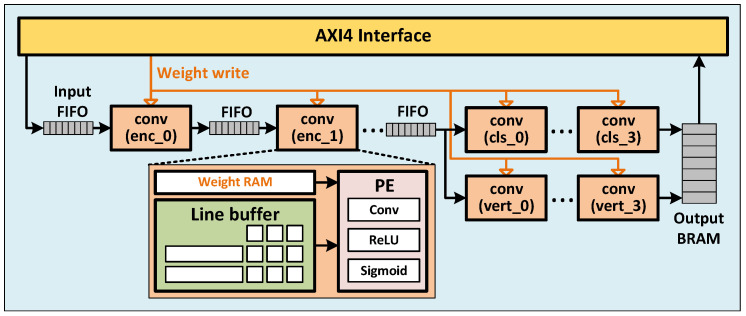
Overall design of the proposed hardware accelerator.

The overall design of our proposed hardware accelerator is presented in Figure 6. Due to the small size of our model, along with the quantized data width, we were able to store the entire model using on-chip block RAMs. Within the design, a 3-D tensor of size C×H×W is treated as a H×W 2-D image, with each pixel being the concatenated bytes across all channels. For example, a 16×128×256 tensor would be stored as a 128×256 image, with each pixel being 16×8=128-bit wide.

Each layer is comprised of a line buffer [47]. This buffer receives input pixel by pixel and lines up the valid sliding window for the subsequent processing element (PE) to compute the multiply–accumulate (MACC) output and the activation. Data flow between each layer is synchronized by intermediate First-in First-out memories (FIFOs), where a layer would read a pixel from the preceding FIFO if it is not empty, and push the output pixels into the succeeding FIFO. All the weights are stored in their respective layer using block RAMs.

This arrangement allows for very highly pipelined computation across layers, as the line buffer of each layer starts receiving data as soon as the first pixel is available from the preceding layer. Because of this, the computation time of the entire model is only slightly longer than the computation time of the first layer. Coupled with the lack of need to fetch data from off-chip DRAM, our accelerator is able to achieve a very high processing speed. The detailed evaluation of the proposed accelerator is presented in Section 5.

### 4.3. Convolution Parallelism

A typical convolution layer with no parallelism would follow a nested loop approach as presented in Algorithm 1, where *L1* iterates through all output pixels, *L2* iterates all output channels, *L3* iterates all input channels and, finally, *L4* iterates through all values in the kernel. To utilize the massive level of parallelism offered by FPGAs, a common approach is to unroll these loops. However, since our model contains many convolution layers with lots of different configurations, a uniform unrolling strategy may become inefficient for certain layers. Because of this reason, several unrolling strategies were explored.
**Algorithm 1** Standard convolution1:**for** (r,c) ← 0 **to** ($H_{y}$,$W_{y}$) **do**
2:  **for** fo ← 0 **to** $C_{y}$ **do**
3:    *Y*[fo][r][c] ←bias[fo]4:    **for** fi ← 0 **to** $C_{x}$ **do**
5:      **for** (kr,kc) ← 0 **to** ($H_{k}$,$W_{k}$) **do**
6:         *Y*[fo][r][c] +=*X*[fi][r+kr][c+kc]*kernel[fo][fi][kr][kc]▹ *L1*▹ *L2* ▹ *L3*▹ *L4* 

The first and most straightforward strategy, named *Incha*, aims to unroll *L3* along with *L4*, as described by Algorithm 2. Assuming *K* is the kernel size (e.g., K=9 for kernel 3×3), $C_{x}$ is the number of input channels, $C_{y}$ is the number of output channels and .* denotes element-wise matrix multiplication, $C_{x}\times K$ multiplications would take place at the same time in parallel, and $C_{y}$ clock cycles would be needed to produce one output pixel.
**Algorithm 2** Incha convolution1:**for** (r,c) ← 0 **to** ($H_{y}$,$W_{y}$) **do**
2:  **for** fo ← 0 **to** $C_{y}$ **do**
3:     *Y*[fo][r][c] ←*sum*(*X*[:][r:r+$H_{k}$-1][c:c+$W_{k}$-1] .* kernel[fo][:][:][:]) +bias[fo]▹ *Incha-L1*▹ *Incha-L2* 

However, for cases where layer $L_{n}$ has $C_{x}=a$ and $C_{y}=b$, followed by layer $L_{n+1}$ with $C_{x}=b$ and $C_{y}=c$, assuming b>a and b>c, layer $L_{n}$ can only produce an output pixel every *b* clocks, thus limiting layer $L_{n+1}$ to receive an input pixel every *b* clocks. The theoretical speed of layer $L_{n+1}$ is one pixel every *c* clocks, but since b>c, such speed cannot be achieved. For such cases, it is more beneficial for layer $L_{n+1}$ to produce a pixel every *b* clocks instead of *c*, thus keeping pace with layer $L_{n}$. Moreover, in this configuration, layer $L_{n+1}$ would only need to perform K×c parallel multiplications instead of K×b, thus consuming less hardware resources. This unroll strategy, named *Outcha*, is shown in Algorithm 3, where *Outcha-L3* is completely unrolled and performed simultaneously. An *Outcha* convolution module performs $C_{y}\times K$ multiplications parallelly and produce an output pixel every $C_{x}$ clock cycles.
**Algorithm 3** Outcha convolution1:**for** (r,c) ← 0 **to** ($H_{y}$,$W_{y}$) **do**2:  *Y*[:][r][c] ←bias[:]3:  **for** fi ← 0 **to** $C_{x}$ **do**4:    **for** fo ← 0 **to** $C_{y}$ **do (unroll)**5:       *Y*[fo][r][c] +=*sum*(*X*[fi][r:r+$H_{k}$-1][c:c+$W_{k}$-1].* kernel[fo][fi][:][:])▹ *Outcha-L1* ▹ *Outcha-L2*▹ *Outcha-L3* 

### 4.4. Convolution Designs

**Figure 7:**
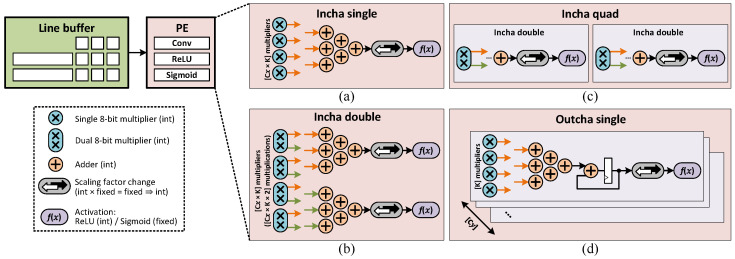
Several versions for convolution module: (**a**) Incha-single, (**b**) Incha-double, (**c**) Incha-quad, and (**d**) Outcha-single.

Based on the unroll strategies presented in Section 4.3, several hardware designs for convolution were made. Based on the analysis presented in Section 4.5, a suitable version is chosen for every layer in the model. Figure 7 shows all the designs for the convolution layer. A convolution module consists of a line buffer that aligns the sliding window for the PE to compute. The PE, depending on which unroll version, contains a configuration of a multiplier array, followed by one or more adder trees to sum up the multiplied products. This multiply–accumulate (MACC) result is then passed into an activation module, which in our model is either ReLU, sigmoid, or linear (no activation). ReLU is computed by a multiplexer and sigmoid is implemented using Piecewise Second-order Approximation [48]. Batch normalization and convolution are merged into one single convolution layer by PyTorch during the quantization process.

For *Incha* strategy, three versions were designed: *single*, *double*, and *quad*. *Incha-single* is designed to perform Algorithm 2 exactly as presented, with an array of $C_{x}\times K$ multipliers followed by an adder tree to sum up all the multiplied products. Utilizing the dual 8-bit multiplier presented in [27], *Incha-double* is designed to double the throughput of *Incha-single* by computing two *Incha-L2* loops while using the same number of DSP48E2 cores. The products are then summed up using two adder trees. Finally, for certain layers, two *Incha-single* modules are combined to create *Incha-quad*.

For *Outcha* strategy (Algorithm 3), only the single version is designed as this strategy is not suitable to have its throughput doubled by the dual 8-bit multiplier. This module consists of $C_{y}$ parallel slices, where each slice contains an array of *K* multipliers, followed by an adder tree and an adder-accumulator. Dual 8-bit multipliers are used by pairs of adjacent slices. This module produces one output pixel for every $C_{x}$ clocks by computing $C_{y}\times K$ multiplications simultaneously.

At synthesis time, a suitable version is chosen for every layer, and the architecture of the layers remains fixed afterward. The analysis details for choosing convolution versions are presented in Section 4.5.

### 4.5. Convolution Designs Analysis

**Figure 8:**
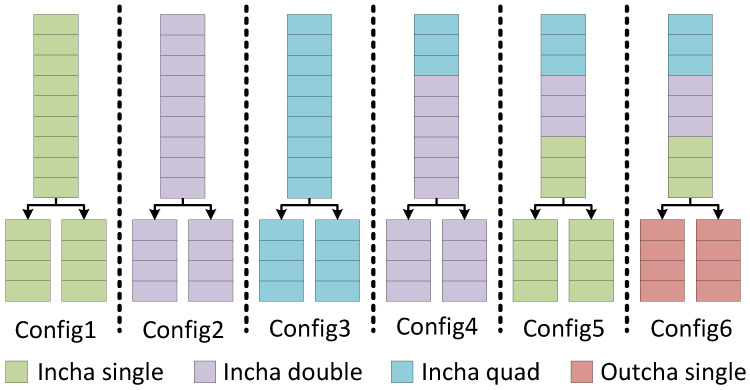
Different hardware configurations for analysis.

**Figure 9:**
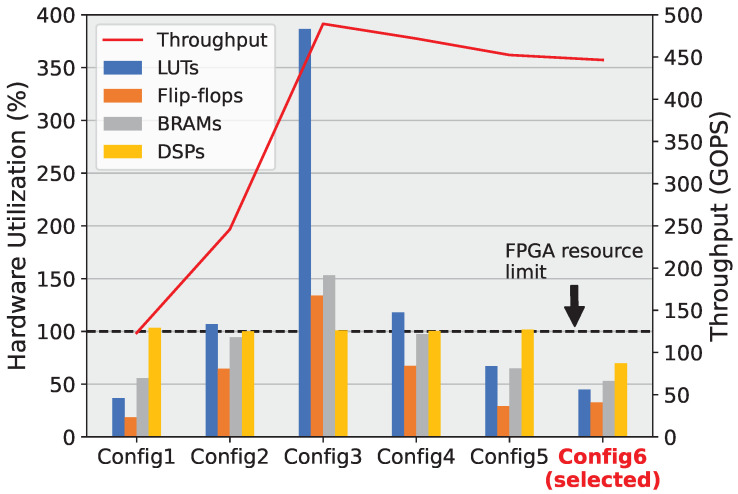
Hardware utilization of different configurations versus throughput.

Due to our proposed model having 17 layers with different configurations, it is imperative that the suitable convolution architecture is applied to each layer to strike the best balance between hardware resources and computation throughput. To find the best combination, we devised and synthesized 6 different configurations to quantitatively analyze. These configurations are synthesized on the Virtex-7 VC707 FPGA, running at 250 MHz. Since most of these configurations are too large to implement on real FPGA, their throughput values are calculated from the simulated clock count. Figure 8 illustrates the different configurations and Figure 9 shows the analysis results.

We first applied the most basic convolution version, *Incha-single*, to every single layer. This is illustrated in Figure 8 as *Config1*. As can be seen in Figure 9, this configuration yields the lowest throughput out of all the analyzed configurations. To increase throughput, we next applied *Incha-double*, which is the version that computes 2 multiplications simultaneously, to all the layers (*Config2* in Figure 8). Looking at Figure 9, it can be seen that throughput has been roughly doubled, at the cost of hardware resources. In fact, this configuration consumes all the DSP cores available of the Virtex-7 FPGA, which forces Vivado to use LUTs (Look Up Tables) to implement the remaining multiplications. This configuration also requires slightly more LUTs than the available number. Lastly, we applied the most resource-intensive convolution version, *Incha-quad*, to all layers (*Config3*). This convolution version computes 4 multiplications in parallel by having 2 *Incha-double* engines inside. As expected, the throughput of *Config3* doubles *Config2* while consuming a massive amount of resources. The number of multipliers required is so much higher than the available DSP cores, that Vivado has to synthesize almost 400% the available LUTs to make up for. Flip-flops and BRAMs also exceed the available amount by 34% and 53%, respectively. With these first 3 configurations, it can be seen that applying the same convolution version to all layers uniformly will result in a very inefficient design, either for throughput or hardware resources. A combination of different versions that results in the best trade-off is necessary.

**Figure 10:**
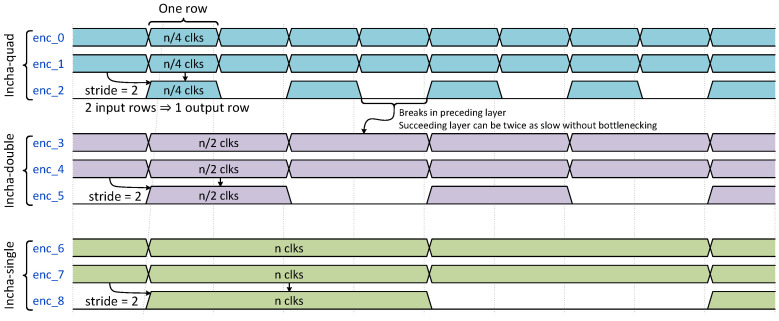
Waveform of encoder layers with different convolution versions: the first three layers use *Incha-quad*, the next three use *Incha-double*, and the last three use *Incha-single*.

One characteristic of QuantLaneNet that can be used to optimize resources without trading performance is the fact that in the encoder stages, every third convolution layer has a stride of 2, while the rest has a stride of 1. These layers only produce an output row for every two input rows received. Because of this, the succeeding layers can afford to compute twice as slowly without introducing a significant bottleneck to the system, as they have a gap every time the preceding layers finish a row. This is illustrated in Figure 10, with the first 3 signals being the valid signals of the first 3 layers. The third layer, *enc_2*, produces one output row for every 2 input rows received from *enc_1* due to it having a stride of 2. Because of this gap, the next layer, *enc_3*, can take twice as long to compute the row it receives from *enc_2* without slowing the entire system down.

To test this hypothesis, we devised *Config4* with the first 3 layers being *Incha-quad*, and all following layers being *Incha-double*, which are twice as slow. From Figure 9, it can be seen that while *Config4* only has a negligible decrease in throughput compared to *Config3*, its resource utilization is improved significantly. This is because the first 3 layers of *Config4* still compute at the same speed as *Config3*, but the rest of the model can still compute twice as slowly without introducing a noticeable performance decrease. This is taken further with *Config5*, where like *Config4*, the first 3 layers are *Incha-quad*, and the next 3 layers are *Incha-double*. However, all the following layers are set to *Incha-single* to be twice as slow as the second triplet of layers. Figure 9 shows that, like *Config4*, *Config5* once again improves resource utilization with only a slight throughput decrease. Finally, as discussed in Section 4.3, the output layers are changed to the more suitable *Outcha-single* version for the final configuration, *Config6*. This is confirmed in Figure 9, where *Config6* consumes the least amount of hardware resource while obtaining 91% the throughput of *Config3*, the configuration with the highest amount of resource utilization. From these analyses, *Config6* is the final configuration chosen for real-world hardware implementation, as it is also the only configuration that fits the resource constraint of the Virtex-7 VC707 FPGA.

## 5. FPGA Implementation, Verification and Results

### 5.1. FPGA Implementation

**Figure 11:**
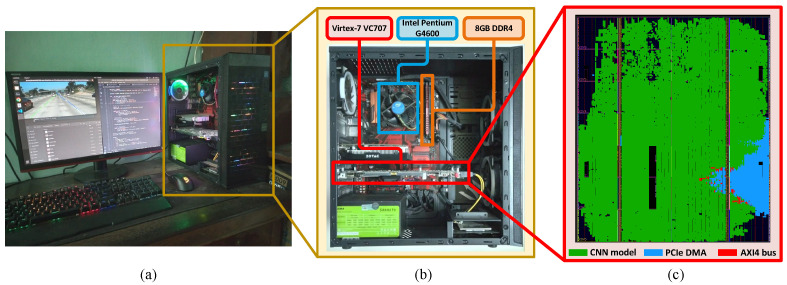
FPGA verification system: (**a**) Full system operation, including results captured on monitor, (**b**) Details of the system, and (**c**) Floorplan of the FPGA chip.

To verify our accelerator design, the Virtex-7 VC707 FPGA is used for practical implementation. The QuantLaneNet core is implemented as an AXI4 IP using hand-written Verilog HDL and the full system is implemented in Vivado using the DMA/Bridge Subsystem for PCI Express IP. The FPGA is connected to an x86-based host PC via the onboard PCIe connector. Details and images of the system are presented in Table 4 and Figure 11, respectively.

The data width of the AXI4 IP is configured to be 64-bit to minimize bus latency and match the data width of the DMA. As there are 8 bytes in 64 bits and 3 bytes in a pixel, there is a mismatch in data widths. Only using 24 bits from the 64-bit is not the most optimized solution. This is because during the data transfer of an image, the bus will not be sending a 64-bit transfer every clock cycle, i.e., there will be empty cycles with no input data during the writing of an image, and the model will need to wait for the next transfer. This can be better optimized by fully utilizing the 64-bit bus, the extra bytes can be stored internally and read by the model in the next clock cycles. When the bus needs to write a new transfer while the model is still processing the bytes from the previous transfer, the bus can be stalled by keeping the AXI_AWREADY signal of the AXI4 bus low.

**Figure 12:**
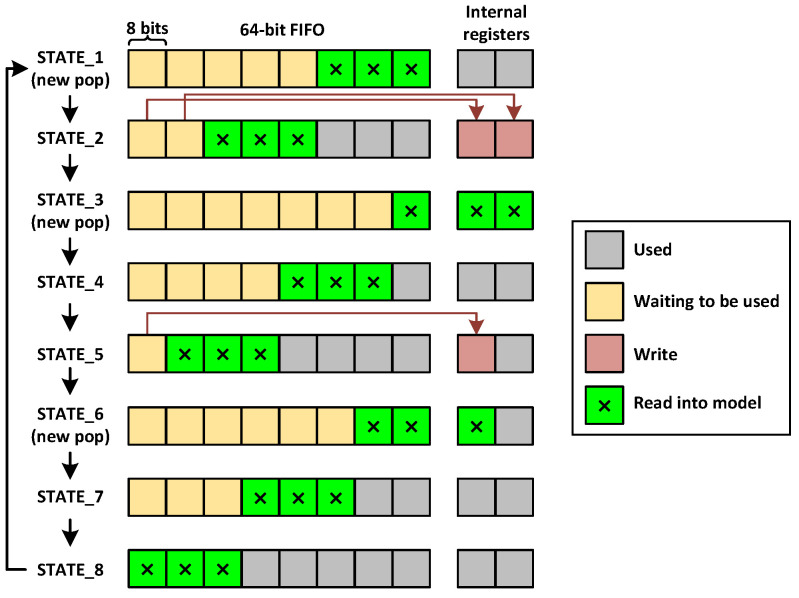
Converting 64-bit writes to 24-bit pixels.

We implement this idea by adding a 64-bit FIFO at the input of the model. The entire image (256×512×3 = 393,216 bytes) is sent via the bus in 8-byte (64-bit) transfers. These transfers are pushed into the FIFO as 64-bit words. The model pops each 24-bit pixel from the FIFO in a mismatch pattern, as shown in Figure 12. The green blocks are the 3 bytes that are read into the model at that clock cycle. If the FIFO is full (due to the bus being written to faster than the model can process), the AXI_AWREADY signal can be kept low to stall the bus. The depth of the FIFO is kept at a moderate size of 512 so that the bus is not bottlenecked while still not consuming too many BRAM blocks.

Using this scheme, an RGB image of size 256×512 only needs 49,152 writes from the processor (256×512×3÷8). Using the burst feature of the AXI4 specification, multiple 64-bit writes can take place in succession, and the empty cycles in between bursts can be made up for by the data already stored in the internal FIFO. This almost completely eliminates bus latency, since there are almost no empty cycles when receiving the pixels, and the first layer can start processing as soon as the first pixel is received (discussed in Section 4.2).

### 5.2. FPGA Verification

**Figure 13:**
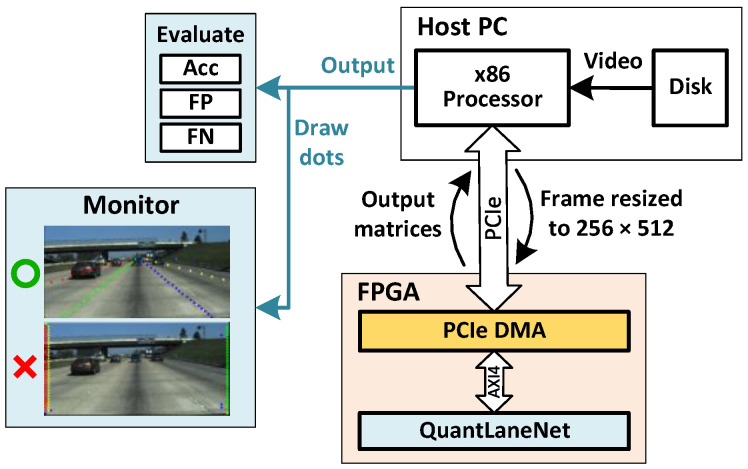
Verification environment.

A software verification environment written in Python is constructed on our x86 PC as illustrated by Figure 13. Raw RGB frames from driving videos stored on the hard disk are extracted and sent to the FPGA to process. This is intended to simulate real-world inputs since in real-world scenarios, inputs from cameras are also read as individual RGB frames. The frames are resized to 256×512 by the CPU before being sent to the FPGA via the PCIe bus. Once the FPGA has finished processing, the output (classification and vertical matrices) is read back into the CPU. To qualitatively verify the results, the output matrices from the FPGA are used to visually draw dots outlining the lane markings on the input frame. The final dot-annotated frame is then presented on the computer monitor. We also evaluate the entire TuSimple dataset using the returned matrices to obtain quantitative metrics.

From the results, we found that the dot-annotated outputs from the FPGA are identical to the outputs from the quantized software model, except for rare cases of slight differences.

For quantitative results, the accuracy evaluation of our hardware accelerator compared to software implementation is presented in Table 5. The accuracy of the hardware implementation (93.43 %) is only reduced by 0.04% compared to the software implementation (93.47 %). This slight reduction is due to the fact that the quantized model in software still contains some floating-point coefficients, but these coefficients are stored as 16-bit fixed-point in the hardware implementation to reduce design complexity. This trade-off in accuracy is acceptable since the accuracy loss is negligible.

### 5.3. FPGA Evaluation

As of the writing of this paper, there have not been any publications with the same exact combination of elements as our work, i.e., CNN model for the task of lane detection implemented on FPGA. Because of this, two studies with similar applications, albeit different datasets, are chosen to provide relative comparisons with the proposed hardware accelerator. The performance, as well as synthesized hardware resource, of our accelerator is presented in Table 6. The results from the studies [26,28] are also shown for comparison. The results of our hardware design are synthesized on the ZCU102 and XCKU115 FPGA for comparisons and on the VC707 FPGA for actual implementation and verification as shown in Figure 11. The framerate of our design is calculated from the average runtime of 100,000 runs. Each runtime starts when the software begins writing the frame to the FPGA and stops when the software has finished reading the output matrices from the FPGA. Power consumption is reported by the Vivado Power Estimator tool.

The results show that our framerates (FPS) and bitrate (Mbps) are much higher than those of [26,28]; meanwhile, our power consumption is less than that of these studies using the same FPGA platforms. Therefore, our system bitrate-based power efficiency (Mbps/W) is much more significant than that of these studies. In addition, as results shown in Table 1, our design takes 0.540 GFLOPs, then throughput reaches 345.6 giga operation per second (GOPS) when our design is implemented on the VC707 FPGA board with the framerate of 640 FPS. This leads to the throughput-based power efficiency reaching 33.52 GOPS/W.

For the hardware resources, when compared to [28], our accelerator design requires a bit more flip-flop and DSP blocks but much fewer LUT and BRAM blocks. When compared to [26], our accelerator design requires more LUT and flip-flop blocks but fewer BRAM and DSP blocks.

## 6. Conclusions

In this work, we present a real-time convolutional neural network for the task of lane detection. Our model minimizes post-processing on the output by utilizing an efficient lane representation format. The model can achieve 93.53% accuracy on the TuSimple dataset while running at 348.34 FPS on the NVIDIA Tesla T4 GPU for image size 256×512. A hardware accelerator is implemented on the Virtex-7 VC707 FPGA to optimize processing speed and power consumption. By utilizing several optimization techniques, such as data quantization and dual 8-bit multiplications on a single multiplier, the hardware accelerator can achieve 640 FPS when running at 250 MHz while consuming only 10.309W. The system throughput and energy efficiency reach 345.6 GOPS and 33.52 GOPS/W, respectively.

## Figures and Tables

**Table 1:** TuSimple dataset evaluation and comparison details.

Model	Input Size	GFLOPs	GPU	FPS	Acc%	FP%	FN%
PINet (1H) [39]	256×512	1.183	RTX 2080 TI	40.0	95.81	5.85	3.30
PINet (2H) [39]		2.032		35.0	96.51	4.67	2.54
PINet (3H) [39]		2.880		30.0	96.72	3.65	2.43
PINet (4H) [39]		3.728		25.0	96.75	3.10	2.50
UFAST-Res18 [37]	288×800	8.436	GTX 1080 TI	322.5	95.87	-	-
UFAST-Res34 [37]		16.959		175.4	96.06		
CondLaneNet-S [38]	320×800	10.300	RTX 2080 TI	220.0	95.48	2.18	3.80
CondLaneNet-M [38]		19.700		154.0	95.37	2.20	3.82
CondLaneNet-L [38]		44.900		58.0	96.54	2.01	3.50
SqueezeNet [46]	288×512	3.558	GTX 1080 TI	111.0	95.27	4.94	4.88
MobileNet_v2 [46]		4.154		71.0	96.34	4.67	5.18
GoogLeNet [46]		4.457		83.0	95.71	4.71	5.26
PolyLaneNet [36]	360×640	1.748	TITAN V	115.0	93.36	9.42	9.33
Ours	256×512	**0.540**	**Tesla T4**	**348.3**	**93.53**	**10.25**	**8.89**

**Table 2:** Accuracy impact of the offset map.

	Acc	FP	FN
W/o offset	93.53%	10.25%	8.89%
W/ offset	93.55%	10.15%	8.79%

**Table 3:** Accuracy of 8-bit quantization.

	Acc	FP	FN
32-bit float	93.53%	10.25%	8.89%
8-bit quant	93.47%	10.38%	9.03%

**Table 4:** FPGA verification system details.

System Details	
CPU	Intel Pentium G4600 @ 3.60 GHz
Memory	8GB DDR4 @ 2400 MHz
Operating System	Ubuntu 20.04.4 LTS
FPGA	Virtex-7 VC707

**Table 5:** Accuracy of hardware accelerator.

	Acc	FP	FN
32-bit float (SW)	93.53%	10.25%	8.89%
8-bit quant (SW)	93.47%	10.38%	9.03%
8-bit quant (HW)	93.43%	10.53%	9.29%

**Table 6:** Hardware implementation evaluation and comparisons.

	RoadNet-RT [28]	ChipNet [26]	Ours		
Device	ZCU102	XCKU115	ZCU102	XCKU115	**VC707**
Precision	8-bit quant	18-bit quant	-	8-bit quant	-
Input size	280 × 960	64 × 180	-	256 × 512	-
Frequency (MHz)	250	350	440	300	**250**
Framerate (FPS)	196.70	79.43	1126.40	768.00	**640.00**
Bitrate (Mbps)	1268.95	21.96	3543.35	2415.92	**2013.27**
Power (W)	-	12.594	12.522	11.194	**10.309**
Pow. Efficiency (Mbps/W)	-	1.74	282.97	215.82	**195.29**
LUTs	260,335 (94.99%)	38,082 (5.74%)	92,728 (33.83%)	98,792 (14.89%)	**136,363** (44.92%)
Flip-flops	115,684 (21.10%)	33,530 (2.53%)	171,680 (31.32%)	171,256 (12.91%)	**198,929** (32.76%)
BRAMs	1340 (73.46%)	1543.0 (71.44%)	494.5 (27.11%)	512.5 (23.73%)	**547.0** (53.11%)
DSPs	1560 (61.90%)	3072 (55.65%)	1957 (77.66%)	1961 (35.53%)	**1957** (69.86%)

## Data Availability

Not applicable.
